# Defining the burden of febrile illness in rural South and Southeast Asia: an open letter to announce the launch of the Rural Febrile Illness project

**DOI:** 10.12688/wellcomeopenres.16393.2

**Published:** 2022-03-10

**Authors:** Arjun Chandna, Rusheng Chew, Nan Shwe Nwe Htun, Thomas J. Peto, Meiwen Zhang, Marco Liverani, Tobias Brummaier, Koukeo Phommasone, Carlo Perrone, Aung Pyae Phyo, Jetsumon Sattabongkot, Wanlapa Roobsoong, Wang Nguitragool, Aninda Sen, Sazid Ibna Zaman, Aye Sandar Zaw, Elizabeth Batty, Naomi Waithira, Mohammad Yazid Abdad, Stuart D. Blacksell, Ladaporn Bodhidatta, James J. Callery, Watcharintorn Fagnark, Witchayoot Huangsuranun, Shayla Islam, Sanchai Lertcharoenchoke, Salisa Lohavittayavikant, Mavuto Mukaka, Vanna Moul, Amit Kumer Neogi, Supalert Nedsuwan, Tiengkham Pongvongsa, Pimsiri Ponsap, Melissa Richard-Greenblatt, William H.K. Schilling, Janjira Thaipadungpanit, Rupam Tripura, Arjen M. Dondorp, Mayfong Mayxay, Nicholas J. White, François Nosten, Frank Smithuis, Elizabeth A. Ashley, Richard J. Maude, Nicholas P.J. Day, Yoel Lubell

**Affiliations:** 1 Centre for Tropical Medicine and Global Health, Nuffield Department of Medicine, University of Oxford, Oxford, UK; 2 Cambodia Oxford Medical Research Unit, Angkor Hospital for Children, Siem Reap, Cambodia; 3 Mahidol-Oxford Tropical Medicine Research Unit, Faculty of Tropical Medicine, Mahidol University, Bangkok, Thailand; 4 Faculty of Medicine, University of Queensland, Brisbane, Australia; 5 Department of Global Health and Development, London School of Hygiene & Tropical Medicine, London, UK; 6 Faculty of Public Health, Mahidol University, Bangkok, Thailand; 7 School of Tropical Medicine and Global Health, Nagasaki University, Nagasaki, Japan; 8 Shoklo Malaria Research Unit, Mahidol-Oxford Tropical Medicine Research Unit, Faculty of Tropical Medicine, Mahidol University, Mae Sot, Thailand; 9 Swiss Tropical and Public Health Institute, Basel, Switzerland; 10 University of Basel, Basel, Switzerland; 11 Lao-Oxford-Mahosot Hospital Wellcome Trust Research Unit, Microbiology Laboratory, Mahosot Hospital, Vientiane, Lao People's Democratic Republic; 12 Mahidol Vivax Research Unit, Faculty of Tropical Medicine, Mahidol University, Bangkok, Thailand; 13 Mahidol University, Bangkok, Thailand; 14 Department of Molecular Tropical Medicine and Genetics, Faculty of Tropical Medicine, Mahidol University, Bangkok, Thailand; 15 Communicable Diseases Programme, BRAC, Dhaka, Bangladesh; 16 Myanmar Oxford Clinical Research Unit, Yangon, Myanmar; 17 Action for Health Department, Battambang, Cambodia; 18 Chiang Rai Provincial Public Health Office, Chiang Rai, China; 19 Savannakhet Provincial Health Department, Savannakhet, Lao People's Democratic Republic; 20 University of Pennsylvania, Pennsylvania, USA; 21 Department of Clinical Tropical Medicine, Faculty of Tropical Medicine, Mahidol University, Bangkok, Thailand; 22 Institute of Research and Education Development, University of Health Sciences, Vientiane, Lao People's Democratic Republic; 23 Harvard TH Chan School of Public Health, Harvard University, Boston, USA; 24 The Open University, Milton Keynes, UK

**Keywords:** Community Health Workers, Etiology, Fever, Primary Health Care, Rural Health, Southeastern Asia, Telemedicine, Western Asia, Village Health Workers

## Abstract

In rural areas of South and Southeast Asia malaria is declining but febrile illnesses still account for substantial morbidity and mortality. Village health workers (VHWs) are often the first point of contact with the formal health system, and for patients with febrile illnesses they can provide early diagnosis and treatment of malaria. However, for the majority of febrile patients, VHWs lack the training, support and resources to provide further care. Consequently, treatable bacterial illnesses are missed, antibiotics are overused and poorly targeted, and patient attendance wanes along with declining malaria.

This *Open Letter* announces the start of a new initiative, the Rural Febrile Illness (RFI) project, the first in a series of projects to be implemented as part of the South and Southeast Asian Community-based Trials Network (SEACTN) research programme. This multi-country, multi-site project will run in Bangladesh, Cambodia, Lao PDR, Thailand, and Myanmar. It will define the epidemiological baseline of febrile illness in nine remote and underserved areas of Asia where malaria endemicity is declining and access to health services is limited.

The RFI project aims to determine the incidence, causes and outcomes of febrile illness; understand the opportunities, barriers and appetite for adjustment of the role of VHWs to include management of non-malarial febrile illnesses; and establish a network of community healthcare providers and facilities capable of implementing interventions designed to triage, diagnose and treat patients presenting with febrile illnesses within these communities in the future.

## Disclaimer

The views expressed in this article are those of the authors. Publication in Wellcome Open Research does not imply endorsement by Wellcome.

## Introduction

The majority of individuals in South and Southeast Asia live in rural areas, often characterised by high levels of poverty and restricted access to healthcare ^1– 3^. Data on causes of disease in these areas to prioritise interventions for scale up are limited. Despite this, there are indications that diseases of an infectious aetiology, which commonly have fever as a presenting symptom and are commonly referred to as ‘febrile illnesses’, account for substantial morbidity and mortality ^4, 5^.

Malaria is the quintessential febrile illness. For decades, empiric antimalarials were recommended for patients presenting with fever ^6^, reflective of the burden associated with this fatal but treatable illness. In many parts of Asia, village health workers (VHWs) and/or village malaria workers (VMWs) were introduced to improve access to treatment as part of wider malaria eradication programmes. Rapid declines in malaria incidence have subsequently been observed ^7^. As a result, in these regions today fever seldom means malaria ^8^, yet the exact cause of the illness often remains unknown, as does optimal management and what becomes of these individuals ^9^.

Falling malaria incidence highlights the inadequacies and fragilities of this vertical approach to healthcare. VHWs/VMWs receive limited training, support, and remuneration, and once malaria has been ruled out, often cannot provide further testing or treatment for their patients. Consequently, treatable bacterial infections are missed ^10, 11^, and antibiotics, if available, are overused and poorly targeted ^11– 13^. Furthermore, the current inability to address non-malarial fever adversely affects uptake of malaria testing: patients have little to gain from having malaria ruled out in areas where it is already rare, but no other care offered for their illness ^14, 15^. This hampers eradication efforts and risks resurgence of drug-resistant malaria ^16, 17^. Encouragingly, it has been shown that the trend of decreasing patient attendance (and malaria testing) can be reversed when the remit of the VHW is extended to other basic healthcare provision ^14^.

There is considerable variation in the training and capabilities of VHWs, and even within the same country there are different types of VHWs, funded by different actors and managed by different institutions. For example, in Lao PDR one such category of VHW is the Village Health Volunteer (VHV), who are managed by the Department of Hygiene and Health Promotion and receive no stipend. They undergo a 10-day training course to support health education, hygiene and sanitation programmes, vaccination, maternal health care, nutrition, the prevention and control of infectious diseases, and the management of revolving drug funds. In contrast, Laotian VMWs are tasked with the diagnosis and treatment of uncomplicated malaria cases, referrals of severe case, distribution of bed nets, and health education/promotion but, unlike VHVs, receive a monthly stipend, usually financed through the Global Fund.

This *Open Letter* announces the start of the Rural Febrile Illness (RFI) project, the first project in the South and Southeast Asian Community-based Trials Network (SEACTN) research programme ^18^. The RFI project will be conducted in Bangladesh, Cambodia, Lao PDR, Thailand, and Myanmar. RFI aims to define the epidemiological baseline of febrile illness in remote and underserved areas of Asia where malaria endemicity has declined and access to health services is limited. The primary objective of the RFI project is to determine the incidence, causes, and outcomes of febrile illness in the rural communities located within the project areas. The longer-term objective of SEACTN is to establish a network of community-based healthcare providers and facilities capable of implementing interventions designed to triage, diagnose, and treat patients presenting with febrile illnesses and other causes of ill health within these communities in the future.

## Project overview

The RFI project is a multi-country, multi-site initiative comprised of a number of prospective observational studies. The project is divided into three key Work Packages (WP) with parallel supporting activities ( Table 1 and Table 2). Work packages A (WP-A) and B (WP-B) will run concurrently and provide the basis for Work package C (WP-C). In brief, WP-A and WP-B, will define the causes of febrile illness in the region, evaluate potential point-of-care tests (POCTs), and describe common pathways for care-seeking. WP-C will focus on the development and pilot implementation of electronic decision support tools (eDSTs), designed using the data from WP-A and WP-B and which will incorporate the use of POCTs found to be promising.

**Table 1.** Key objectives of the Rural Febrile Illness project.

Overall	
1	Determine the incidence, causes and outcomes of febrile illness in nine rural areas of Bangladesh, Cambodia, Lao PDR, Thailand, and Myanmar
2	Establish a network of community healthcare providers and facilities, capable of implementing interventions designed to triage, diagnose and treat patients presenting with febrile illnesses within these communities in the future
Work Package A	
1	Develop electronic data collection tools for patients presenting to village health workers and primary health centres with febrile illnesses in the study areas
2	Determine the incidence and outcomes of febrile illnesses amongst patients presenting to village health workers and primary health centres in the study areas
3	Describe and understand health status and health-seeking behaviour for febrile illnesses in the study areas
4	Describe common causes of mortality and events immediately preceding death in the study areas
5	Map the geographic location, accessibility, treatment availability and workforce capacity of health facilities within and nearby the study areas
Work Package B	
1	Describe the causes and outcomes of febrile illnesses amongst patients presenting to sentinel rural health facilities in the study areas
2	Determine the diagnostic performance of host biomarkers to distinguish bacterial from viral infections amongst patients presenting with febrile illnesses
3	Determine the prognostic performance of host biomarkers to identify patients with febrile illnesses at risk of severe outcomes
Work Package C	
1	Complete stakeholder analyses to identify the opportunities, barriers and appetite for adjustment of the role of community healthcare providers to include management of non-malarial causes of febrile illness
2	Model the cost-effectiveness of different combinations of interventions to improve the management of febrile illness in the study areas
3	Develop and pilot electronic decision-support tools and point-of-care tests that can assist community healthcare providers in their assessment, triage and treatment of patients with febrile illnesses

**Table 2.** Rural Febrile Illness project activities planned at each of the study sites. AHEAD, Action for Health Development; CCRU, Chiang Rai Clinical Research Unit; MAM, Medical Action Myanmar; MVRU, Mahidol Vivax Research Unit; SMRU, Shoklo Malaria Research Unit; SPHO, Savannakhet Provincial Health Office.

Site	Implementing partner	Febrile illness in primary care	Verbal autopsies	Household health surveys	Febrile illness in higher-level health facilities	Stakeholder mapping and analysis
**Cox’s Bazaar & Bandarban** **districts, Bangladesh**	BRAC	✓	✓	✓	✓	✓
**Battambang & Pailin** **provinces, Cambodia**	AHEAD	✓	✓	✓		✓
**Kachin state, Myanmar**	MAM	✓	✓	✓	✓	✓
**Kayin state, Myanmar**	SMRU	✓				
**Tak province, Thailand**	SMRU				✓	
**Tak province, Thailand**	MVRU	✓				
**Yala province, Thailand**	MVRU	✓				
**Chiang Rai province, Thailand**	CCRU	✓	✓	✓	✓	
**Savannakhet province, Lao** **PDR**	SPHO	✓	✓		✓	✓

Sites will commence recruitment for WP-A and WP-B at different times given their unique circumstances, in particular the Covid-19 control measures in place at each site. WP-A commenced at the first site (Savannakhet, Lao PDR) in August 2021, and we anticipate completing all work packages by October 2023. Detailed protocols for individual components of the RFI project are available at www.seactn.org.

### Work Package A (WP-A)

In collaboration primarily with VHWs, we will develop and deploy electronic data collection tools to capture the incidence, presenting syndromes, and outcomes of febrile illness amongst individuals presenting to the most peripheral level of the health system. In Lao PDR, community healthcare providers working at primary health centres (PHCs) rather than VHWs will implement the project, as utilisation of VHWs is currently low in this country ^2^; a similar approach will be taken in Chiang Rai province of Thailand.

Data collected from VHWs and PHCs will be complemented by parallel health status and health-seeking behaviour surveys, verbal autopsies, and village and health facility mapping projects, designed to gain a comprehensive understanding of the burden of febrile illness and access to care in the areas served by these health facilities and providers. We will also perform targeted aetiological investigations in a subset of patients presenting to WP-A providers and facilities.

### Work Package B (WP-B)

WP-B activities will be concentrated around two types of health facilities which provide higher-level care (health clinics and/or hospitals) in each of the sites in Bangladesh, Lao PDR and Myanmar, as well as two of the four sites in Thailand. These higher-level facilities are located within (or nearby) the same geographical area as the VHWs and PHCs selected for WP-A. More extensive aetiological investigations, as well as assays of host biomarkers, will be performed in patients with febrile illnesses attending these facilities.

### Work Package C (WP-C)

In WP-C, we will draw on the data collected in WP-A and WP-B to create temporally- and spatially-explicit eDSTs. These eDSTs will be designed to assist community health workers (both VHWs and healthcare providers at PHCs) in their assessment, triage, and treatment of patients presenting with febrile illnesses in rural and remote areas. The data from WP-A and WP-B will also be used to identify the most high-impact and cost-effective POCTs that could be included within the eDSTs, as well as appropriate delivery mechanisms. Subsequent deployment and health system integration will be informed by stakeholder analyses in the same settings to better understand the opportunities, barriers, and appetites for adjustment of the roles of VHWs and other community healthcare providers to include management of non-malarial causes of febrile illness.

### Study sites and implementing partners

**Figure 1.**
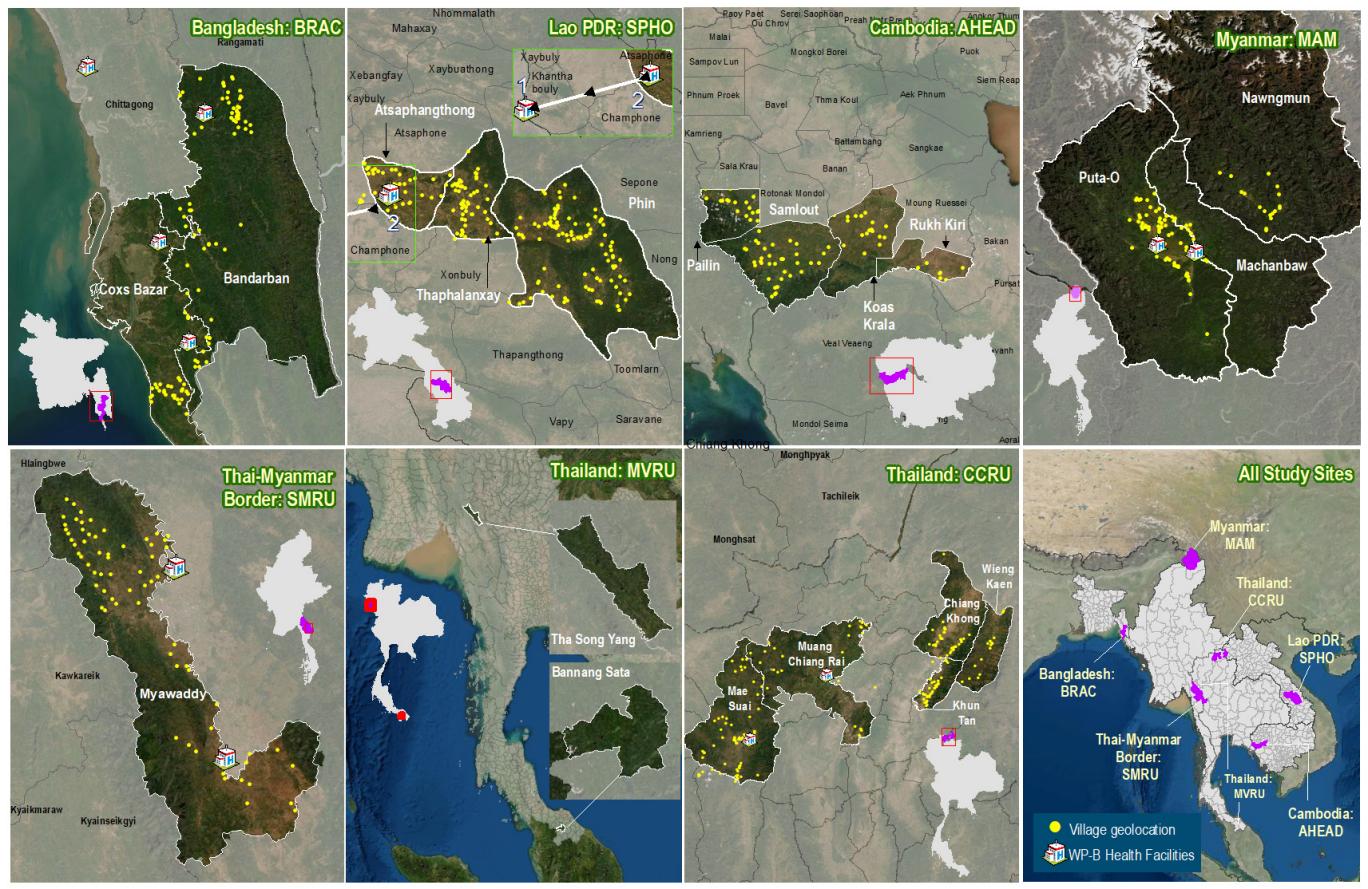
Study areas with selected villages and health facilities. AHEAD, Action for Health Development; CCRU, Chiang Rai Clinical Research Unit; MAM, Medical Action Myanmar; MVRU, Mahidol Vivax Research Unit; SMRU, Shoklo Malaria Research Unit; SPHO, Savannakhet Provincial Health Office; WP-B, Work Package B. Due to access difficulties related to the ongoing Covid-19 pandemic and civil unrest in Myanmar, all activities at the Myanmar (MAM) site are suspended until further notice. WP-B activities are currently not planned for the Cambodia (AHEAD) and Thailand (MVRU) sites.

Strong, long-standing partnerships have been leveraged to plan and implement this multi-disciplinary project, across at least 620 villages in nine rural regions of Bangladesh, Cambodia, Lao PDR, Thailand, and Myanmar ( Figure 1). Site selection was based on these pre-existing collaborative relationships and village selection was delegated to the partner organisations, who are best placed to know the needs of, and challenges facing, the communities they serve.

In Bangladesh the project will be implemented in partnership with BRAC, one of the largest non-governmental development organisations in the world. As of September 2021, 130 villages have been selected and their corresponding local health facilities (Upazila Health Complexes, District Hospitals, and Medical College Hospitals) identified in Cox’s Bazar and Bandarban districts. Recruitment for both WP-A and WP-B is expected to begin at the end of 2021.

The project in Cambodia involves WP-A (including the verbal autopsy and household health surveys described below), and is a collaboration with the National Center for Parasitology, Entomology and Malaria Control (CNM), the Provincial Health Departments of Battambang and Pailin provinces, and Action for Health Development (AHEAD), a civil society organisation working to improve health in rural communities. The study will take place in western Cambodia in up to 90 villages from Pailin and Battambang provinces, and is co-funded by the Global Fund through its Regional Artemisinin-resistance Initiative (RAI) grant. In Cambodia, both PHCs and VMWs, the latter of which have a narrower scope of clinical practice than VHWs, will be involved in patient recruitment.

Two study sites are planned in Myanmar. In northern Kachin State, 89 villages and two health clinics (Himalaya Clinic I and II), have been selected across three townships (Puta-O, Machanbaw, and Nawngmun), where the non-governmental healthcare organisation Medical Action Myanmar (MAM) operates a network of VHWs and rural health clinics. The second site is in Kayin state straddling the Thai-Myanmar border, where the Shoklo Malaria Research Unit (SMRU) supervises a large community health worker programme focused on malaria elimination. Overall, 130 villages and two health clinics (Wang Pha and Mawker Thai) across the townships of Hlaingbwe, Myawaddy, and Kawkareik are planned for selection. Given the location of the SMRU programme, this site is expected to commence study activities in the clinics, which are located on the Thai side of the border, in the last quarter of 2021, notwithstanding the civil unrest in Myanmar.

In southern Lao PDR, 28 PHCs serving 160 villages within three districts (Atsaphangthong, Thaphalanxay, and Phin), as well as Atsaphangthong district hospital and Savannakhet provincial hospital have been selected. The project will be conducted in partnership with Savannakhet Provincial Health Office (SPHO). Recruitment for WP-A commenced in August 2021 and is ongoing; recruitment for WP-B is expected to begin at the end of 2021.

Two sites in Thailand will commence recruitment for WP-A in the final quarter of 2021. The first of these is located in Chiang Rai province and will be run in collaboration with the Chiang Rai Clinical Research Unit (CCRU) and the Chiang Rai Provincial Public Health Office. At this site, five PHCs will recruit patients from 26 villages. The second site covers 82 villages in Yala and Tak provinces, and will be run in collaboration with the Mahidol Vivax Research Unit (MVRU), which in turn leverages the existing government malaria control programme infrastructure consisting of village malaria workers (VMWs), malaria posts, malaria clinics, community clinics, and PHCs.

## Work Package A

### Electronic data collection tools

Electronic data collection forms have been developed using the CommCare platform (Cambridge, MA) and loaded on to mobile Android tablets. The tablets, accompanied by solar chargers where necessary, will be distributed to all participating VHWs/VMWs and PHCs. Their feedback will inform iteration of the data collection forms following a human-centred design approach ^19^. Once finalised, the tablets will be used to capture data from patients with febrile illnesses who attend the VHWs/VMWs and PHCs.

Data will be securely uploaded in real-time. In areas without mobile internet, uploads will occur periodically during visits by the local implementing partner (BRAC, AHEAD, MAM, SMRU, SPHO, CCRU, and MVRU). The aim is to develop a data stream from source to web-based interface with near real-time geospatial mapping of the incidence and outcomes of febrile illness. A detailed Data Management Plan is available on request from the Mahidol-Oxford Tropical Medicine Research Unit (MORU) Data Access Committee.

### Digital health education materials

Training materials have been developed in partnership with DigitalMedic and serve three primary purposes:

- To sensitize communities to the project aims and objectives;
- To support in-person training of the VHWs and PHC workers, focusing on ensuring accurate syndromic classification of febrile illnesses, reliable collection of clinical data and biological samples, and confidence in operating the electronic data collection tool;
- To improve patient understanding of the health problems the project aims to address.

### Community engagement activities

Prior to the initiation of the study, the local implementing partner will meet with villagers, village leaders, and relevant local authorities to explain the project rationale, aims, and planned activities, using digital educational material as outlined above. Community consent will be sought for the collection of basic demographic and syndromic data using the same electronic data collection tool from all patients attending VHWs/VMWs and PHCs to better understand the health needs of the community and inform future directions of the SEACTN.

### Determining the incidence and outcomes of febrile illnesses at the primary care level

All patients who attend participating VHWs/VMWs and PHCs will be screened, and those with a febrile illness who provide consent will be enrolled. For this study, acute febrile illness is defined as a documented fever (≥37.5°C axillary) or hypothermia (<35.5°C) at presentation and/or history of fever in the last 24 hours. Enrolment is age-unrestricted, will be consecutive, and is planned for 18 months at all sites. Based on current attendances we estimate that we will capture approximately 100,000 febrile illness episodes across all study sites.

The primary health worker will use the electronic data collection tool to record a limited set of clinical and demographic data as well as the result of the malaria rapid diagnostic test (mRDT). No changes to patient management will occur as a result of the study, except that the health worker will be prompted to consider referral and/or inform the operations team of the implementing partner if any danger signs are elicited, subject to the specific context at each site ^20– 22^. The participant will be followed up 28 days later to determine the outcome of their illness. As far as possible, follow-up will be conducted face-to-face, with participants reimbursed for travel costs. However, given the remoteness of some of the study villages, follow-up may be conducted by telephone if the participant finds it too onerous to attend in person. This interval was chosen because many of the diagnostic investigations involve paired serology, thus an adequate amount of time needs to have elapsed to allow for clinically significant changes in antibody titres. Furthermore, as many of the villages are relatively inaccessible, participants may find it too onerous to re-attend for follow-up if the interval is short.

In participants outside the neonatal age range a capillary blood sample (finger- or heel-prick) will be collected at the same time as the mRDT and stored as a dried blood spot (DBS) on filter paper. In a subset of participants (at least 20,000 illness episodes) a convalescent DBS will be collected at the one-month follow-up. Targeted molecular and serological investigations ( Table 3) will be performed on an initial set of 10,000 illness episodes to determine the likely aetiologies of the febrile illnesses, with remaining samples stored for future targeted analyses. VHWs/VMWs will be trained in dried blood spot collection prior to patient enrolment, for which the relevant site-specific ethical approvals have been obtained.

**Table 3.** Aetiological investigations to be performed in the RFI project, including specimen type, target pathogen, diagnostic platform and laboratory location ^25– 27^. *Dengue NS1 testing will be performed on the acute (D0) sample only. **Blood cultures will be performed in-country at a quality-assured laboratory close to the WP-B health facility. MORU, Mahidol Oxford Tropical Medicine Research Unit; MVRU, Mahidol Vivax Research Unit; NS1, non-structural protein 1; PHC, primary health centre; NPS, nasopharyngeal swab; RDT, rapid diagnostic test; VHW, village health worker; VMW, village malaria worker.

Setting	Specimen	Target pathogen	Laboratory location and diagnostic platform		
			MORU/MVRU PCR (Day 0)	MORU Paired serology (Day 0 and Day 28)	On site (Day 0)
**Work Package A:** **VHWs/VMWs and PHCs**	Capillary blood	*Plasmodium* spp.	✓		RDT
		Dengue *	✓	✓	
		Chikungunya	✓	✓	
		Pan-Alphavirus	✓		
		Pan-Flavivirus	✓	✓	
		*Orientia tsutsugamushi*	✓	✓	
		*Rickettsia typhi*		✓	
		*Rickettsia* spp.		✓	
		*Leptospira* spp.		✓	
**Work Package B:** **Rural secondary care** **or higher-level health** **facilities**	Capillary blood	*Plasmodium* spp.			RDT or microscopy
	Venous blood	Dengue	✓	✓	
		Chikungunya	✓	✓	
		Pan-Alphavirus	✓		
		Pan-Flavivirus	✓	✓	
		*Orientia tsutsugamushi*	✓	✓	
		*Rickettsia* spp.	✓	✓	
		*Leptospira* spp.	✓	✓	
		16S rRNA (eubacteria)	✓		
		Bacterial bloodstream infections			Blood culture **
	NPS	Respiratory pathogens	✓		

### Household health surveys

We will conduct health status and health-seeking behaviour surveys to understand pathways to care for individuals with febrile illnesses. A better appreciation of how care for acute illness is currently sought in these settings will enable us to determine the overall burden of febrile illness in the study areas, and identify the best options for potential intervention in the future.

A cross-sectional household survey will be conducted targeting villages participating in WP-A, with households residing in the study area eligible for the survey. Two-stage cluster-randomized sampling of the households will be performed. In the first stage, villages of each study site will be selected; and in the second stage households located in the selected villages will be selected. All members of the selected households will be invited to participate in the survey. Participants will not be selected based on presence or absence of symptoms or diseases, and participation in WP-A does not affect the eligibility for survey participation.

Community-level data, such as estimated vaccine coverage, availability of water, sanitation, and hygiene (WASH) facilities, and assessments of indoor air quality, will be gathered to help contextualise the project’s findings.

### Verbal autopsies

Little information exists on the febrile (and non-febrile) causes of mortality in the study areas ^23^. In collaboration with researchers from the University of Toronto, we have adapted the World Health Organization’s (WHO) Verbal Autopsy (VA) tool to conduct electronic VA questionnaires for all deaths that occur in SEACTN villages in Bangladesh, Cambodia, Lao PDR, the Kachin state site in Myanmar, and the Chiang Rai site in Thailand ^24^. Understanding the common causes of mortality and the circumstances that surround death will enable us to identify targets for interventions that can be implemented and evaluated within the SEACTN programme in the future.

### Village and health facility mapping

A thorough understanding of local healthcare infrastructure is essential to direct referrals to higher-level care appropriately. Villages, transport networks, and health facilities in the study areas will be mapped using field collection and satellite imagery, and detailed profiles created of the study villages including population statistics, communication and transport systems, health services and campaigns, physical environment, and socioeconomic metrics. WHO’s AccessMod 5 software will be used to estimate travel times from the study villages to health facilities and identify potential gaps in local service provision. In addition to informing how future interventions can be deployed within the SEACTN programme, we will provide this information to health system planners and policy makers to help identify where new health facilities could be provided and existing services strengthened to achieve highest impact.

## Work Package B

### Determining the incidence and outcomes of febrile illnesses at higher-level health facilities

To gain a comprehensive understanding of the causes of febrile illness in the region, we will recruit a cohort of patients attending two sentinel higher-level health facilities within five of the project areas (Bangladesh, Lao PDR, Chiang Rai province in Thailand, the Thai-Myanmar border, and northern Kachin state in Myanmar). The information from these cohorts will be combined with the aetiological data from WP-A, where we are limited to collecting low-volume DBS specimens from a subset of participants as the remote locations preclude collection of samples from all villages and prevent maintenance of a cold chain.

All patients aged >28 days who attend the health facilities with a febrile illness will be screened, and those who provide consent will be enrolled. Six hundred participants (inpatients and outpatients) will be enrolled in each age stratum (>28 days to <5 years; ≥5 years to <15 years; and ≥15 years with no upper age limit), across all participating health facilities within a single project area (i.e. 1,800 participants in each area). Recruitment is planned for a minimum of 12 continuous months at each site to ensure seasonality is adequately captured.

Baseline data will be recorded including demographics, anthropometrics, presenting syndrome, vital and other clinical signs, duration of illness, and any care sought for the illness thus far. At the Bangladesh, Lao PDR, Chiang Rai and Thai-Myanmar border sites blood cultures will be collected and the results provided to the treating clinical teams.

Venous blood samples and nasopharyngeal swabs will be collected and transported to MORU’s central laboratories in Bangkok, Thailand where molecular and serological aetiological investigations ( Table 3) will be performed. Novel molecular techniques such as target enrichment sequencing have also been validated against existing molecular diagnostics and will be used to investigate a broader range of pathogens than can be detected by the pathogen-specific tests ^28^. Additional investigations for participants with specific syndromes (for example, urine, pus, throat swab, and/or cerebrospinal fluid cultures) will be considered, subject to feasibility at each of the facilities.

Admitted participants will be followed-up daily during their admission. All participants will be followed-up on day 2 (in person or via telephone) and asked to return to the health facility 28 days after enrolment to determine the outcome of their illness. A venous blood sample will be collected at this time for convalescent serological testing.

### Identification of clinical features and host biomarkers that distinguish bacterial from viral infections in febrile patients

The venous blood samples collected from patients attending the rural health facilities will also be used to quantitatively measure host biomarkers that reflect immune activation and endothelial dysfunction. We will use this information, together with the baseline clinical data and results of the aetiological investigations, to construct diagnostic algorithms that can distinguish bacterial from viral infections ^29– 32^. Host biomarkers feasible for measurement using POCTs will be prioritised, in order to augment the development of algorithms that can be used to guide antimicrobial prescribing in resource-limited primary care settings.

### Development of prognostic clinical prediction models for febrile patients at risk of severe outcomes

We will also use the host biomarker data, in conjunction with the baseline clinical and day 2 and 28 outcome data, to derive prognostic algorithms (clinical prediction models) to identify patients at risk of severe outcomes, which could be used to guide referral decisions from community healthcare settings to higher-level care ^33– 38^. The baseline clinical data, host biomarker panels, and outcome definitions have been harmonised with a parallel study (NCT04285021) to facilitate data sharing and external validation of the prediction models.

Host biomarkers will be selected on the basis of biological plausibility ^39^, systematic review of the literature ^29, 33^, and the results of ongoing prospective studies ^40, 41^. The aim is to identify and test biomarkers that discriminate bacterial from viral infections, and those that are implicated in final common pathways to sepsis and severe febrile illness independent of microbial aetiology. In community settings, it is often not possible to obtain an aetiological diagnosis for the vast majority of patients with febrile illness during routine clinical care and, hence, our aim is to develop diagnostic and prognostic algorithms that can be applied at the syndromic level by limited-skill health workers and be used to inform common clinical management decisions e.g., whether to prescribe an antibiotic, which antibiotic to prescribe, and whether the patient requires referral to higher-level care. Biomarkers will be measured retrospectively using central laboratory-based assays (ELISAs and multi-analyte panels) ^42^. The results will not be available to providers in real time during the initial observational phases of the project.

## Work Package C

### Stakeholder mapping and analyses

Throughout the RFI project, key stakeholders will be engaged and interviewed on the topic of expanding the remit of VHWs and/or other community healthcare providers to include management of non-malarial febrile illnesses. Stakeholders will include policymakers and managers within the Ministries of Health of the respective countries; representatives of international and national donor organisations; individuals and organisations responsible for the implementation and supervision of VHW and other community-based health programmes; and VHWs/VMWs and community members.

During the interviews, information and views will be collected about operational challenges, opportunities and policy bottlenecks concerning the expansion of VHW programmes, with particular attention to the key issues of health system integration, capacity, and sustainability. After an initial set of interviews with seed informants selected after a stakeholder mapping exercise, additional interviewees will be identified through snowball sampling. Collected material will be analysed thematically, while interim outputs such as matrix tables or position maps will be constructed to understand better the roles and interests of different stakeholder categories, their resources, and their levels of support for the expansion of VHW/VMW programmes. The findings will also inform participatory development of the eDSTs, as described below.

### Economic evaluation of interventions that could be deployed in the SEACTN to improve the management of febrile illness

We will draw on the data and findings from WP-A and WP-B to develop economic models to assess the cost-effectiveness of different interventions, or combinations of interventions, to improve the management of febrile illness suitable for assessment via clinical trials in subsequent projects within the SEACTN. Analyses will be conducted on standalone interventions such as the implementation of POCTs ^43^, as well as multi-layered approaches integrating regional data on causes of illness within eDSTs and alongside POCTs ^44^. The outputs of the cost-effectiveness analyses will also be provided to relevant governmental and non-governmental actors to support the planning and scale-up of national programmes.

### Development of eDSTs for use by rural community healthcare providers

**Figure 2.**
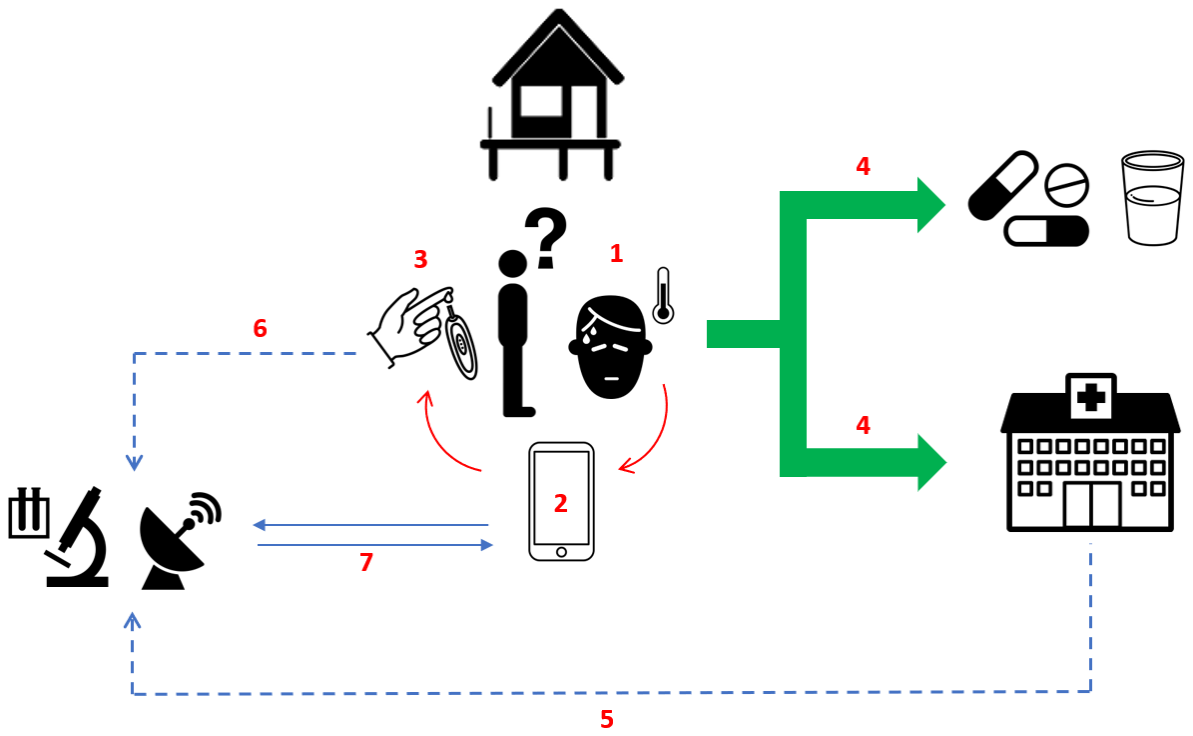
Overview of the long-term ambition for the management of febrile illnesses within SEACTN. (1) Patient with febrile illness presents to village health worker (VHW). (2) Simple clinical algorithm pre-loaded on mobile device helps VHW assess patient. (3) VHW performs point-of-care test (POCT) if recommended by algorithm. (4) VHW decides between community-based treatment or referral for higher-level care. (5) Regional health facilities (sentinel nodes) periodically provide data from patients attending with febrile illnesses (including patients referred by VHWs working within SEACTN). (6) Proportion of samples collected from febrile patients at the time POCTs are performed, stored on filter paper and transported to reference laboratories. (7) Data from (5) and (6) integrated with data from (2) to periodically update clinical algorithms to reflect seasonal and longitudinal changes in febrile illness landscape.

The results from these modelling assessments, and the data from WP-A and WP-B, will be used to design bespoke, spatially-explicit eDSTs that can assist community healthcare providers in their assessment, triage, and treatment of patients with febrile illnesses ( Figure 2). POCTs will be but one component of the eDSTs, which will be dynamic, algorithm-based, and developed using artificial intelligence principles using the rich datasets resulting from WP-A and WP-B.

The eDSTs will be loaded on to the mobile tablets that the VHWs/VMWs and PHC workers are already familiar with using for data collection. Interactive training modules will be developed, again in partnership with DigitalMedic, that will include educational content on the common causes of febrile illness in the region, as well as instructional information on how to use the new eDSTs and any relevant POCTs. User feedback will be sought and the eDSTs iterated using a human-centred design process ^19^. Once finalized, the eDSTs will be put forward for evaluation in future projects to be conducted within the SEACTN.

## Dissemination of findings

Interim findings from relevant aspects of the project (for example, aggregate results of the aetiological investigations for febrile illness and the VA study) will be periodically summarised and fed back to the local communities via village leaders and local authorities. This will allow important information to be actioned in a time-sensitive manner, whilst preserving the confidentiality of individual participants.

The final results generated from this study will be disseminated to the key stakeholders identified during the stakeholder mapping exercises and the study communities (via the same community engagement forums used to launch the project) in both English and local languages. The results will also be shared with the scientific community via peer-reviewed publications and conference presentations.

## Limitations

Studying febrile illness at the most peripheral level of a health system provides a unique opportunity to influence the course of a patient’s illness at their first contact with formal health services. This is particularly important in settings where regulation of facilities, providers, and treatments is often lacking or inadequately enforced. However, working at this level of the health system also poses certain challenges.

We are limited to collecting low-volume DBS specimens from patients attending VHWs/VMWs and PHC workers in WP-A. The aetiological yield of these specimens may be low, for example due to confounding caused by incidental micro-organism detection and false-negative results due to small sample volumes. However, they are feasible for collection in large numbers and, without attempts to understand the causes of fever in rural areas of the region, meaningful improvements in the management of febrile illness will likely remain elusive. The limited clinical diagnostic capabilities of the primary health workers may also introduce error in the formulation of syndromic diagnoses. To mitigate these risks, we will recruit cohorts of patients attending sentinel health facilities within these areas in WP-B, where collection of a wider range of specimens will permit more extensive aetiological investigations. Furthermore, the yield from the DBS specimens will be monitored and reviewed by the RFI Study Management Group. Depending on the results, DBS assays may be expanded or replaced with an alternate strategy.

Expansion of the roles of VHWs/VMWs is both feasible and impactful ^14^. However, there is a limit to the number of roles that these essentially semi-skilled laypeople can be expected to fulfil without adequate recognition, supervision, and remuneration ^45^. Stakeholder engagement, planned throughout the RFI project, will be crucial to understand the feasibility of long-term adjustments to the VHW/VMW role.

## Conclusion

The RFI project aims to better understand and quantify the burden of febrile illness, its aetiologies, and the manner in which it affects people living in some of the most underserved areas of South and Southeast Asia, all on a scale which has not been attempted before. We will collect information to understand better and predict the outcomes of patients with febrile illnesses based on a multitude of factors, which will form the basis for interventions within the SEACTN in the future.

The foundational infrastructure established by the RFI project will include a network of upskilled and supported community healthcare providers; user-friendly electronic data collection tools; functioning biological specimen collection, transport, and diagnostic pipelines; and robust data management systems for near real-time geospatial mapping of the incidence and outcomes of patients with febrile illness. SEACTN will be well-positioned to support ongoing surveillance of febrile illnesses in the region, enabling earlier detection of disease outbreaks and the regular updating of treatment algorithms in response to seasonal and longitudinal changes in the regional febrile illness landscape.

## Data availability

No data are associated with this article.
