# Supplementary material for: Defining the burden of febrile illness in rural South and Southeast Asia: an open letter to announce the launch of the Rural Febrile Illness project
Source: Wellcome Open Res. 2022 Mar 10;6:64. Originally published 2021 Mar 26. [Version 2] doi: 10.12688/wellcomeopenres.16393.2 (PMC8080974; doi:10.12688/wellcomeopenres.16393.2)
Supplement: Peer review history

# Peer review: Reviewer response for version 2

**DOI:** 10.21956/wellcomeopenres.19385.r49117

**Published:** 2022-04-19

**Authors:** Emily Ciccone

**Affiliations:** 1 Division of Infectious Diseases, School of Medicine, University of North Carolina, Chapel Hill, NC, USA

No further comments.

Does the article adequately reference differing views and opinions?

Yes

Are all factual statements correct, and are statements and arguments made adequately supported by citations?

Yes

Is the Open Letter written in accessible language?

Yes

Where applicable, are recommendations and next steps explained clearly for others to follow?

Not applicable

Is the rationale for the Open Letter provided in sufficient detail?

Yes

Reviewer Expertise:

I am an early career physician scientist primarily focused on improving the management of pediatric febrile illness in sub-Saharan Africa (specifically Uganda and Malawi). My current projects study the use of rapid diagnostics, including biomarker and pathogen-specific testing, at peripheral health centers with limited laboratory capacity and by VHWs to improve antibiotic stewardship among children with acute respiratory illness.

I confirm that I have read this submission and believe that I have an appropriate level of expertise to confirm that it is of an acceptable scientific standard.

# Peer review: Reviewer response for version 1

**DOI:** 10.21956/wellcomeopenres.18033.r43501

**Published:** 2021-04-30

**Authors:** Emily Ciccone

**Affiliations:** 1 Division of Infectious Diseases, School of Medicine, University of North Carolina, Chapel Hill, NC, USA

This open letter describes plans for a large, multi-country project focused on describing the etiology and improving the management of febrile illness in rural areas of South and Southeast Asia. This ambitious work is incredibly important as the widespread availability of malaria testing and treatment has led to a decline in malaria incidence, leaving most febrile illnesses undifferentiated. This diagnostic uncertainty leads to inappropriate and inadequate treatment, including the overuse of antibiotics, which drives antimicrobial resistance, a major threat to global public health.

 In this article, the authors clearly outline their objectives and plans for implementation of the initiative, and the maps in Figure 1 distinctly show the varied locations of the included study sites. I also appreciated their acknowledgment of the challenges of working in rural areas with VHW providers, and think that there was sufficient explanation and justification of their plans. The continual reassessment of the feasibility of expanding the VHW role through stakeholder engagement itself will provide interesting information that will shape the evolution of such programs in response to the changing epidemiology of the conditions that VHW treat.

 A few minor comments related to each Work Package are as follows:

WP-A

1. Please clarify the patient population that will be included. As the VHWs will be the primary providers that are included in the study, I assume the patient population will be children under 5 years of age, but would recommend clearly stating the target study population and if there are any differences in the age groups that will be enrolled at each site.
2. How will follow-up assessments be done? In other words, will they be in-person visits or phone calls? I assume in-person for at least a portion as convalescent DBS are being collected, but some clarification here would be useful.
3. How was 28 days chosen for the follow-up interval? This seems to be quite long for acute febrile illnesses. A brief justification of how this interval was chosen would be helpful.
4. Will the health-seeking behaviour surveys be conducted for all participants in WP-A or a subset? If a subset, how with the sampling be done?

WP-B

1. Again, a bit more clarification about age group would be helpful here - will any adults be enrolled or does the >/= 15 age group only extend up to 18 years of age?
2. Would be interested in more detail regarding the host biomarker testing. Specifically, which tests are planned? Will the results of POCT be available to providers in real time?

WP-C

1. I found Figure 2 to be a bit confusing, particularly the group of icons in the center. Perhaps it would be helpful to include a few more arrows demonstrating the progression of the initial assessment (presentation --> mobile algorithm --> POCT --> location of care decision).

A few stylistic/grammatical things to consider are as follows:

- Could delete word "residing" in last paragraph of the introduction as communities themselves don't res
- Spell out BRAC when it is first mentioned in the Study sites and implementing partners section.
- Should be "local facilities have been identified" in same paragraph
- Remove the parentheses around "combinations of" in the paragraph about economic evaluation of interventions in WP-C section.

Does the article adequately reference differing views and opinions?

Yes

Are all factual statements correct, and are statements and arguments made adequately supported by citations?

Yes

Is the Open Letter written in accessible language?

Yes

Where applicable, are recommendations and next steps explained clearly for others to follow?

Not applicable

Is the rationale for the Open Letter provided in sufficient detail?

Yes

Reviewer Expertise:

I am an early career physician scientist primarily focused on improving the management of pediatric febrile illness in sub-Saharan Africa (specifically Uganda and Malawi). My current projects study the use of rapid diagnostics, including biomarker and pathogen-specific testing, at peripheral health centers with limited laboratory capacity and by VHWs to improve antibiotic stewardship among children with acute respiratory illness.

I confirm that I have read this submission and believe that I have an appropriate level of expertise to confirm that it is of an acceptable scientific standard.

# Response

**Published:** 2021-12-14

**Authors:** Arjun Chandna

**Affiliations:** Mahidol-Oxford Tropical Medicine Research Unit, Thailand

**WP-A: Please clarify the patient population that will be included. As the VHWs will be the primary providers that are included in the study, I assume the patient population will be children under 5 years of age, but would recommend clearly stating the target study population and if there are any differences in the age groups that will be enrolled at each site.**

 For WP-A, there are no age restrictions in the inclusion criteria, which will be uniform at all sites.

 The relevant sentence has been amended to reflect this: “Enrolment is age-unrestricted, will be consecutive, and is planned for 18 months at all sites.”

**WP-A: How will follow-up assessments be done? In other words, will they be in-person visits or phone calls? I assume in-person for at least a portion as convalescent DBS are being collected, but some clarification here would be useful.**

 As far as possible, follow-up assessments will be conducted in person, and participants will have their travel costs reimbursed for attendance at follow-up visits. However, given the remoteness of some villages, telephone follow-up will be done if participants are not able to present in person.

 The following has been added to clarify this point: “As far as possible, follow-up will be conducted face-to-face, with participants reimbursed for travel costs. However, given the remoteness of some of the study villages, follow-up may be conducted by telephone if the participant finds it too onerous to attend in person.”

**WP-A: How was 28 days chosen for the follow-up interval? This seems to be quite long for acute febrile illnesses. A brief justification of how this interval was chosen would be helpful.**

 This interval was chosen because many of the diagnostic investigations involve paired serology, thus an adequate amount of time needs to have elapsed to allow for clinically significant changes in antibody titres. Furthermore, as many of the villages are relatively inaccessible, participants may find it too onerous to re-attend for follow-up if the interval is short. The preceding sentences have been added verbatim to the manuscript to explain our reasoning.

**WP-A: Will the health-seeking behaviour surveys be conducted for all participants in WP-A or a subset? If a subset, how with the sampling be done?**

 The following has been added to the section on Household Health Surveys to address this query: “A cross-sectional household survey will be conducted targeting villages participating in WP-A, with households residing in the study area eligible for the survey. Two-stage cluster-randomized sampling of the households will be performed. In the first stage, villages of each study site will be selected; and in the second stage households located in the selected villages will be selected. All members of the selected households will be invited to participate in the survey. Participants will not be selected based on presence or absence of symptoms or diseases, and participation in WP-A does not affect the eligibility for survey participation.”

**WP-B: Again, a bit more clarification about age group would be helpful here - will any adults be enrolled or does the >/= 15 age group only extend up to 18 years of age?**

 Yes, adults will be enrolled.

 To avoid misunderstanding, the relevant sentence has been amended to “Six hundred participants (inpatients and outpatients) will be enrolled in each age stratum (>28 days to <5 years; ≥5 years to <15 years; and ≥15 years with no upper age limit), across all participating health facilities within a single project area (i.e. 1,800 participants in each area).”

**WP-B: Would be interested in more detail regarding the host biomarker testing. Specifically, which tests are planned? Will the results of POCT be available to providers in real time?**

 We have added the following paragraph to the section on biomarkers, which addresses test selection and availability of POCT results: “Host biomarkers will be selected on the basis of biological plausibility, systematic review of the literature, and the results of ongoing prospective studies. The aim is to identify and test biomarkers that are implicated in the final common pathways to sepsis and severe febrile illness and shared across a range of microbial aetiologies. In community settings, it is not possible to obtain an aetiological diagnosis for the vast majority of patients with febrile illness during routine clinical care and, hence, our aim is to develop ‘pathogen agnostic’ diagnostic and prognostic algorithms that can be applied at the syndromic level by limited-skill health workers and be used to inform common clinical management decisions e.g., whether to prescribe an antibiotic, which antibiotic to prescribe, and whether the patient requires referral to higher-level care. Biomarkers will be measured retrospectively using central laboratory-based assays (ELISAs and multi-analyte panels). The results will not be available to providers in real time during the initial observational phases of the project.”

**WP-C: I found Figure 2 to be a bit confusing, particularly the group of icons in the center. Perhaps it would be helpful to include a few more arrows demonstrating the progression of the initial assessment (presentation --> mobile algorithm --> POCT --> location of care decision).**

 We have added some additional arrows to the group of icons in the centre to clarify the expected sequence of events during a patient encounter. We hope that this, in combination with the legend of the figure, will provide a clear infographic summary of the overall conceptual framework for the medium- to long-term ambition of SEACTN.

**General: Could delete word "residing" in last paragraph of the introduction as communities themselves don't res (sic)**

 ‘Residing’ has been changed to ‘located’.

**General: Spell out BRAC when it is first mentioned in the Study sites and implementing partners section.**

 BRAC is now the official name of the organization and is not an acronym. We have reflected this in the manuscript.

**General: Should be "local facilities have been identified" in same paragraph.**

 This sentence has now been modified for clarity.

**General: Remove the parentheses around "combinations of" in the paragraph about economic evaluation of interventions in WP-C section.**

 The sentence has been amended to “We will draw on the data and findings from WP-A and WP-B to develop economic models to assess the cost-effectiveness of different interventions, or combinations of interventions, to improve the management of febrile illness suitable for assessment via clinical trials in subsequent projects within the SEACTN.”

# Peer review: Reviewer response for version 1

**DOI:** 10.21956/wellcomeopenres.18033.r43499

**Published:** 2021-04-23

**Authors:** Teresa B. Kortz

**Affiliations:** 1 Department of Pediatrics, University of California, San Francisco (UCSF), San Francisco, CA, USA

Overall, a clear, well-written letter. This is an important topic and the proposed initiative has the potential to positively impact child health.

 I may have missed it, but how is this being funded and what is the timeline?

**Abstract**

- Clear, no comments.

**Introduction**

- Suggest describing village health workers some more. For example, who are they, what role do they play, how do they improve access to treatment...
- Are the declines in malaria incidence related to VHWs? That is how I interpreted the second paragraph.

**Project overview**

- What is the proposed timeline for this project and the various work packages?

**Study sites and implementing partners**

- Was there a site selection process and if so, what was it? Was there an effort to recruit a certain type or number of community?
- How is "febrile illness" defined for the sake of study inclusion?
- Which "host biomarkers feasible for measurement using POCTs, will be prioritised"?

**Dissemination/Limitations/Conclusion**

- No specific comments.

Does the article adequately reference differing views and opinions?

Yes

Are all factual statements correct, and are statements and arguments made adequately supported by citations?

Yes

Is the Open Letter written in accessible language?

Yes

Where applicable, are recommendations and next steps explained clearly for others to follow?

Partly

Is the rationale for the Open Letter provided in sufficient detail?

Yes

Reviewer Expertise:

sepsis, severe febrile illness, pediatric critical care in resource-variable settings, host prognostic biomarkers, fever etiology, resource utilization

I confirm that I have read this submission and believe that I have an appropriate level of expertise to confirm that it is of an acceptable scientific standard.

# Response

**Published:** 2021-12-14

**Authors:** Arjun Chandna

**Affiliations:** Mahidol-Oxford Tropical Medicine Research Unit, Thailand

**Introduction: Suggest describing village health workers some more. For example, who are they, what role do they play, how do they improve access to treatment...**

 Please see the response above to the similar question asked by Reviewer 1, which addresses this comment in detail.

**Introduction: Are the declines in malaria incidence related to VHWs? That is how I interpreted the second paragraph.**

 This is partly correct – VHWs were introduced as part of wider malaria control programmes, but were not the sole reason for the decline in malaria incidence.

 We have clarified this point as follows: “In many parts of Asia, village health workers (VHWs) and/or village malaria workers (VMWs) were introduced to improve access to treatment as part of wider malaria eradication programmes. Rapid declines in malaria incidence have subsequently been observed.”

**Project overview: What is the proposed timeline for this project and the various work packages?**

 The project has an anticipated completion date of June 2023. Given site-specific circumstances, especially COVID-19 restrictions, sites will commence recruitment for the work packages at different times, with the first site in Laos having started recruitment for WP-A in August 2021.

 This has now been included as follows: “Sites will commence recruitment for WP-A and WP-B at different times given their unique circumstances, in particular the Covid-19 control measures in place at each site. WP-A will commence at the first site in August 2021, and we anticipate completing all work packages by June 2023. Detailed protocols for individual components of the RFI project are available at www.seactn.org.”

**Study sites and implementing partners: Was there a site selection process and if so, what was it? Was there an effort to recruit a certain type or number of community?**

 We have mentioned that the SEACTN project leverages on strong, long-standing partnerships, but have elaborated on this further to include details on site and village selection by adding the following sentence: “Site selection was based on these pre-existing collaborative relationships and village selection was delegated to the partner organizations, who are best placed to know the needs of, and challenges facing, the communities they serve.”

**How is "febrile illness" defined for the sake of study inclusion?**

 Acute febrile illness is defined as a documented fever (≥37.5°C axillary) or hypothermia (<35.5°C) at presentation and/or history of fever in the last 24 hours. This has now been added to the manuscript.

**Which "host biomarkers feasible for measurement using POCTs, will be prioritised"?**

 Please see response to the comment on biomarkers from Reviewer 3 below, which answers this question comprehensively.

# Peer review: Reviewer response for version 1

**DOI:** 10.21956/wellcomeopenres.18033.r43297

**Published:** 2021-04-20

**Authors:** Samuel Akech

**Affiliations:** 1 Health Services Unit, KEMRI-Wellcome Trust Research Programme, Nairobi, Kenya

The open letter communicates about a series of planned studies in several study sites in Asia to define aetiology of febrile illnesses, develop and implement point of care tests (POCTs) to differentiate bacterial versus viral infections, develop electronic decision support (eDST), and implement the POCTs and eDSTs using community health workers (village health workers and primary health care workers) for referral and treatment. In this letter, the authors have summarised linked pieces of research projects that are grouped into 3 work packages with several specific objectives. It is therefore expected that sufficient detail would be missing in a project that has several components. However, the ultimate success of this project as described would a successful implementation of the eDST, POCTs, and referral/treatment by the Community Health Workers (CHWs).

**Comments:**

 A brief description of training and capabilities of CHWs in the study settings will assist readers to gain insights into prospects of successful implementation. What other tasks do the CHWs already have? Will therefore be detailed implementation science conducted to understand implementation challenges for the project and is there a conceptual framework for the project implementation? It would be good to briefly mention how the implementation is expected to work at the level of CHWs who are quite central. Are the CHWs remunerated, what is their motivation, how much are researchers working with policy makers in the study countries-some insights will be good. Are the village health workers collecting heel/thumb prick samples? Is this something that they are permitted to do in this setting-this could be briefly explained.   

 Are there examples of treatments that CHWs would be allowed to give based on results of POCTs? If POCTs suggests a bacterial infection, it may be harmful to give an antibiotic and send the patient home since causes of a positive bacterial test could be varied e.g., UTI, sepsis, intra-abdominal infections (especially in adults), and it is difficult to imagine an antibiotic that can be given by CHWs that would cover all potential causes without need for a referral. In such cases, eDST would be configured to trigger a referral or no referral.

 It would seem more logical if aetiological study and development of POCTs were done at earlier phases and then promising POCTs implemented plus eDST using CHWs. Can a diagram for sequence of activities be included?;

Does the article adequately reference differing views and opinions?

Yes

Are all factual statements correct, and are statements and arguments made adequately supported by citations?

Yes

Is the Open Letter written in accessible language?

Yes

Where applicable, are recommendations and next steps explained clearly for others to follow?

Not applicable

Is the rationale for the Open Letter provided in sufficient detail?

Yes

Reviewer Expertise:

Malaria, sepsis, clinical trials, paediatrics, epidemiology, severe childhood illness

I confirm that I have read this submission and believe that I have an appropriate level of expertise to confirm that it is of an acceptable scientific standard.

# Response

**Published:** 2021-12-14

**Authors:** Arjun Chandna

**Affiliations:** Mahidol-Oxford Tropical Medicine Research Unit, Thailand

**A brief description of training and capabilities of CHWs in the study settings will assist readers to gain insights into prospects of successful implementation. What other tasks do the CHWs already have? Will therefore be detailed implementation science conducted to understand implementation challenges for the project and is there a conceptual framework for the project implementation? It would be good to briefly mention how the implementation is expected to work at the level of CHWs who are quite central. Are the CHWs remunerated, what is their motivation, how much are researchers working with policy makers in the study countries-some insights will be good.**

 There is a high degree of variability in the roles, capabilities, and remuneration of village health workers, even within the same country. We have provided further details on these points:

 “There is considerable variation in the training and capabilities of VHWs, and even within the same country there are different types of VHWs, funded by different actors and managed by different institutions. For example, in Lao PDR one such category of VHW is the Village Health Volunteer (VHV), who are managed by the Department of Hygiene and Health Promotion and receive no stipend. They undergo a 10-day training course to support health education, hygiene and sanitation programmes, vaccination, maternal health care, nutrition, the prevention and control of infectious diseases, and the management of revolving drug funds. In contrast, Lao VMWs are tasked with the diagnosis and treatment of uncomplicated malaria cases, referrals of severe case, distribution of bed nets, and health education/promotion but, unlike VHVs, receive a monthly stipend, usually financed through the Global Fund.”

 Implementation research will, indeed, be conducted to identify and understand the relevant challenges and opportunities beginning with the stakeholder analysis component of the project, details of which are provided later in the open letter.

**Are the village health workers collecting heel/thumb prick samples? Is this something that they are permitted to do in this setting-this could be briefly explained.**

**

 Yes, they are – this has been approved by the relevant ethics committees. All participating village health workers will be trained in study procedures prior to site activation.

 The following sentence has been added to clarify this point: “VHWs/VMWs will be trained in dried blood spot collection prior to patient enrolment, for which the relevant site-specific ethical approvals have been obtained.”

**Are there examples of treatments that CHWs would be allowed to give based on results of POCTs? If POCTs suggests a bacterial infection, it may be harmful to give an antibiotic and send the patient home since causes of a positive bacterial test could be varied e.g., UTI, sepsis, intra-abdominal infections (especially in adults), and it is difficult to imagine an antibiotic that can be given by CHWs that would cover all potential causes without need for a referral. In such cases, eDST would be configured to trigger a referral or no referral.**

 As mentioned, the ultimate aim of WP-C is to develop bespoke electronic algorithm-based decision support tools covering both diagnosis and management. To avoid management decisions based solely on POCT results, the algorithms will have the ability to aid primary health workers in formulating syndromic diagnoses and selecting the antibiotic regimen most likely to be of clinical benefit. POCTs will be but one component in these algorithms, and distinguishing bacterial from viral infection merely one of the several uses envisaged for them.

 We have modified the relevant sentences to reflect this: “These eDSTs will be designed to assist community health workers (both VHWs and healthcare providers at PHCs) in their assessment, triage, and treatment of patients presenting with febrile illnesses in rural and remote areas. The data from WP-A and WP-B will also be used to identify the most high-impact and cost-effective point-of-care tests (POCTs) that could be included within the eDSTs, as well as appropriate delivery mechanisms.”

 “POCTs will be but one component of the eDSTs, which will be dynamic, algorithm-based, and developed using artificial intelligence principles using the rich datasets resulting from WP-A and WP-B.”

**It would seem more logical if aetiological study and development of POCTs were done at earlier phases and then promising POCTs implemented plus eDST using CHWs. Can a diagram for sequence of activities be included?**

 Yes, this is indeed the sequence of activities in this project – WP-A and WP-B will run concurrently. In these work packages, the causes of febrile illness will be defined, potential POCTs evaluated, and eDSTs developed. WP-C will focus on the implementation of these eDSTs, which will incorporate the use of POCTs found to be promising.

 We have added the following to the Project Overview section, which sets out the timeline succinctly: “In brief, WP-A and WP-B, will define the causes of febrile illness in the region, evaluate potential point-of-care tests (POCTs), and develop electronic decision support tools (eDSTs). WP-C will focus on the implementation of these eDSTs, which will incorporate the use of POCTs found to be promising.”
